# A Novel *In Vitro* Sensing Configuration for Retinal Physiology Analysis of a Sub-Retinal Prosthesis

**DOI:** 10.3390/s120303131

**Published:** 2012-03-06

**Authors:** Kyo-in Koo, Sangmin Lee, Jang Hee Yee, Sang Baek Ryu, Kyung Hwan Kim, Yong Sook Goo, Dong-il Dan Cho

**Affiliations:** 1 Inter-university Semiconductor Research Center, Automation System Research Institute, School of Electrical Engineering and Computer Science, Seoul National University, Seoul 151-744, Korea; E-Mails: kkin76@snu.ac.kr (K.K.); sangmlee@snu.ac.kr (S.L.); 2 Department of Physiology, Chungbuk National University, Cheongju 361-763, Korea; E-Mail: tim1981-yjh@hanmail.net; 3 Department of Biomedical Engineering, Yonsei University, Wonju 220-710, Korea; E-Mails: satisfaction@hanmail.net (S.B.R.); khkim0604@yonsei.ac.kr (K.H.K.)

**Keywords:** sensing configuration, microelectrode array, sub-retinal prosthesis

## Abstract

This paper presents a novel sensing configuration for retinal physiology analysis, using two microelectrode arrays (MEAs). In order to investigate an optimized stimulation protocol for a sub-retinal prosthesis, retinal photoreceptor cells are stimulated, and the response of retinal ganglion cells is recorded in an *in vitro* environment. For photoreceptor cell stimulation, a polyimide-substrate MEA is developed, using the microelectromechanical systems (MEMS) technology. For ganglion cell response recording, a conventional glass-substrate MEA is utilized. This new sensing configuration is used to record the response of retinal ganglion cells with respect to three different stimulation methods (monopolar, bipolar, and dual-monopolar stimulation methods). Results show that the geometrical relation between the stimulation microelectrode locations and the response locations seems very low. The threshold charges of the bipolar stimulation and the monopolar stimulation are in the range of 10∼20 nC. The threshold charge of the dual-monopolar stimulation is not obvious. These results provide useful guidelines for developing a sub-retinal prosthesis.

## 1. Introduction

Retinal degeneration is a prevalent disease that can lead to total blindness in people; it refers to a group of diseases with similar symptoms, including age-related macular degeneration (ARMD) and retinitis pigmentosa (RP) [1]. It is estimated that the ARMD affects 1/4 people over 64 [2], and the RP influences one in four thousand people [3]. Unfortunately, pharmaceutical treatments to restore vision have not yet been discovered, but there are at present a number of treatments to attempt to slow down the progression of certain forms of retinal degeneration [4].

Despite the degeneration of the outer nuclear layer in these cases of retinal degeneration blindness, there are reports that the inner retinal cells (78.4%) and retinal ganglion cells (29.7%) partially survive [5–7]. Many groups have researched retinal prostheses, which stimulate the surviving cells for vision restoration [8–14]. Early chronic implants have allowed some patients to detect motion and discern simple objects [15–17]. However, optimized stimulation protocols (electrode size, electrode spacing, stimulation signal pulse shape, stimulation signal pulse duration, and stimulation signal pulse frequency) have not been assessed yet. The principal reason is the complexity of visual perception pathway, from the retina through the optic nerve to the visual cortex.

On the technical side, single metal wires or conical shaped electrodes have been employed for extracellular stimulation and recording [18–21]. Some researchers used electrodes for stimulation, but the response was recorded using a whole cell patch clamp technique [22,23]. Other researchers investigated multi-electrode stimulation and recording in isolated retinas [24–26]. These three experimental configurations correspond to *in vitro* epi-retinal configurations. A photodiode-based approach for a sub-retinal configuration has also been reported, which eliminates the need for a camera [11].

In this paper, photoreceptor cell stimulation and retinal ganglion cell (RGC) response recording using two microelectrode arrays (MEAs) is presented. A retinal patch is placed on a conventional glass-substrate MEA, with ganglion cells facing down. Then a developed polyimide-substrate MEA is placed on top, facing photoreceptor cells. This sensing configuration enables multi-stimulation and multi-recording *in vitro.* This is a sub-retinal prosthesis configuration. Then, the response patterns of monopolar, bipolar, and dual-monopolar stimulation methods are investigated. The threshold current of each different stimulation method is also studied.

## 2. Methods

### 2.1. Retinal Tissue Preparation

All animal use protocols were approved by the Institutional Animal Care Committee of Chungbuk National University (permit number: CA-25). Rabbits are dark adapted overnight prior to being anesthetized with intramuscular injection of xylazine (20 mg/kg) and ketamine (200 mg/kg) sufficient to extinguish forefoot withdrawal reflex. Retinas of dark-adapted New Zealand white rabbits (male, approximately 2 kg) are isolated after anesthetization. For the retinal tissue preparation, the method of in [27] is utilized. Briefly, after the eyeball is enucleated, the retina is isolated and cut into patches of approximately 5 × 5 mm size. Subsequently, the retinal patches are placed in artificial cerebrospinal fluid (ACSF) bubbled with 95% O_2_ and 5% CO_2_ at 32 °C.

### 2.2. Configuration of the Prepared Retinal Tissue and MEAs

**Figure 1.**
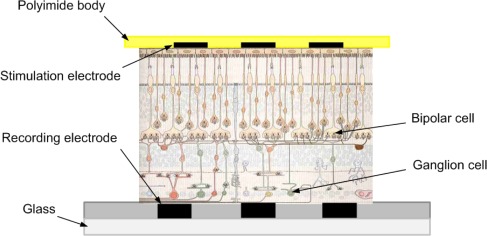
The schematic of the electrophysiological configuration.

For the stimulation and recording configuration, the ganglion cell layer part is placed on the glass recording MEA (MEA 200/30, Multi Channel Systems MCS GmbH, Reutlingen, Germany), and then the stimulating MEA is attached on top, to the photoreceptor cell layer part, as shown in Figure 1.

**Figure 2.**
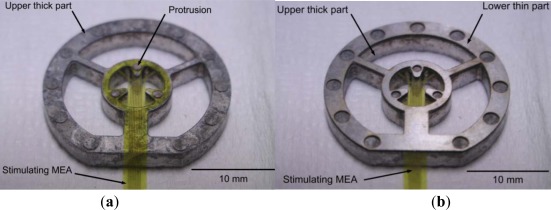
Fixture for stimulation MEA. (**a**) Upper thick part with MEA on polyimide (shown upside down). (**b**) Lower thin part placed on top to thick part (shown upside down).

**Figure 3.**
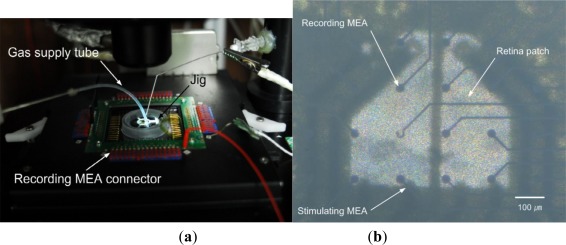
Experimental setup. (**a**) Interconnection between the MEAs in the camber and the data acquisition system. (**b**) Sensing part showing recording MEA, isolated retina and the stimulation MEA, which corresponds to center part of Figure 2. (Note that the microscope is focused at the recording MEA and the other layers are out of focus).

The stimulating MEA is microfabricated using polyimide, Ti, and Au. In general, polyimide films float in water. For fixation of the developed MEA on polyimide, a jig is fabricated, using aluminum (the lower thin part) and stainless steel (the upper thick part), as shown in Figure 2. The MEA on polyimide is placed between the lower thin part and the upper thick part. The lower thin part is 200 μm thick, and protrusion in the upper thick part is 150 μm thick. Therefore, if the lower thin part is used, the height from the recording MEA to the stimulating MEA is 200 μm. If the lower thin part is not used, the height from the recording MEA to the stimulating MEA is about 150 μm. The polyimide isolation layer is very thin (∼8 μm). The stimulating MEA with the customized jigs dips into the ACSF medium in the recording MEA chamber, where the isolated retinal patch is attached. Note that these pieces have a center opening for alignment. Figure 3 shows the experimental setup.

### 2.3. Microelectrode Array

The recording MEA has 60 circle-shaped TiN microelectrodes (30 μm diameter) insulated by Si_3_N_4_ on a glass substrate. The recording MEA is spaced in an 8 × 8 square-type grid layout, except for every vertex. Each microelectrode is separated 200 μm apart from each other. The impedance of the recording microelectrode is 50 kΩ at 1 kHz.

**Figure 4.**
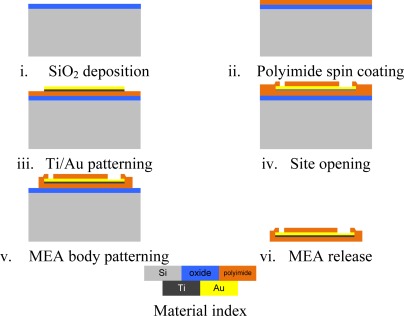
The fabrication process flow stimulating MEA.

The stimulating MEA is fabricated with polyimide (PI-2525, HD Micro Systems, Tokyo, Japan), Ti, and Au at the Inter-university Semiconductor Research Center (Seoul, Republic of Korea) using the microelectromechanical systems (MEMS) technology. All materials are biocompatible [28,29]. A stimulating microelectrode has a 30 μm diameter, and a reference microelectrode has a 70 μm diameter. They are separated 600 μm apart from each other. The fabrication process is described in Figure 4. First, SiO_2_ is deposited on Si substrate for releasing device at the final step. Polyimide (8 μm) is spin coated and cured as a lower base layer. Titanium and gold are deposited and patterned for the microelectrodes and conductive lines. Then, polyimide (8 μm) is spin coated again, and patterned for the site opening openings and base shaping. Finally, using hydrofluoric acid solution, the polyimide-substrate MEA is released from the substrate.

### 2.4. Electrical Stimulating and Recording Experiments

The SRG 4002 (Multi Channel Systems MCS GmbH) is connected to the developed MEA. The MEA 60 recording system (Multi Channel Systems MCS GmbH) is used for electrical potential recording. The data acquisition system consists of a RS-232 interface for the synchronization of stimulation and recording, and an integrated 60-channel MEA 1060 amplifier (gain: 1200, band pass: 10–300 Hz). Control software (MC Rack, Multi Channel Systems MCS GmbH) synchronizes and controls all equipment.

**Figure 5.**
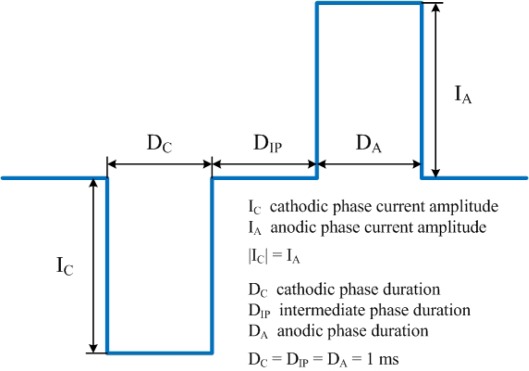
Cathodic-first biphasic symmetric square-shaped current pulse with 1 ms duration and 1 ms intermediate phase.

Cathodic-first biphasic symmetric square-shaped current pulses (Figure 5) with a 1 ms duration and a 1 ms intermediate phase are used to the stimulating MEA. These biphasic symmetric current pulses are commonly used for the safety of tissues and microelectrodes [30]. In this paper, amplitudes of 5∼200 μA are experimented. Both cases are experimented with a universal reference (monopolar case) and a local reference (bipolar case), respectively. Electrical response is measured from the recording MEA with a sampling rate of 25 kHz/channel. All measurement values are the averaged values of 50 repetitions.

### 2.5. Data Analysis Method

Recordings within 5 ms of stimulation are usually obscured by stimulation artifacts. An average subtraction technique is utilized for artifact removal to restore the spike trains within ∼5 ms post-stimulation [25]. First, recorded waveforms near the stimulation onset are identified as two distinct classes, with or without the evoked RGC action potentials. From multiple waveforms which did not seem to contain the evoked action potentials, average waveforms of the stimulation artifact are estimated. The waveforms of RGC spikes within ∼5 ms after stimulation onset can be recovered by subtracting the obtained averaged stimulation artifact from the single trial waveforms containing action potentials.

The subtracted data are transformed into single unit spike trains using spike sorting software (Offline Sorter, Plexon Inc., Dallas, TX, USA). Post-stimulation time histograms are constructed for characterization of the evoked RGC spikes. Number of the evoked RGC spikes is counted to analyze the modulation of the RGC response with respect to the pulse amplitude. The single unit spike trains are converted to a firing rate time series by counting the numbers of spikes within 500 ms bins. For quantification of accuracy of the input information representation within the RGC response, similarities between the time series of pulse amplitude variation and the firing rate time series are computed by calculating correlation coefficients of the two time series using MATLAB (MathWorks, Natick, MA, USA).

## 3. Results

We performed seven experiments (three bipolar stimulations, three monopolar stimulations and one dual-monopolar stimulation), using three retinal patches isolated from one rabbit. The response tendency of the evoked RGC spike number is similar among the three retinal patches with respect to the stimulation method and the stimulation current amplitude. However, the spatial response distribution of the evoked RGC spikes is difficult to find among the three retinal patches. In this chapter, representative results from one retinal patch are described in detail, due to space limitation. The discussion section describes complete results with all three patches. In addition, additional data are appended.

### 3.1. Single Stimulation (Monopolar and Bipolar)

**Figure 6.**
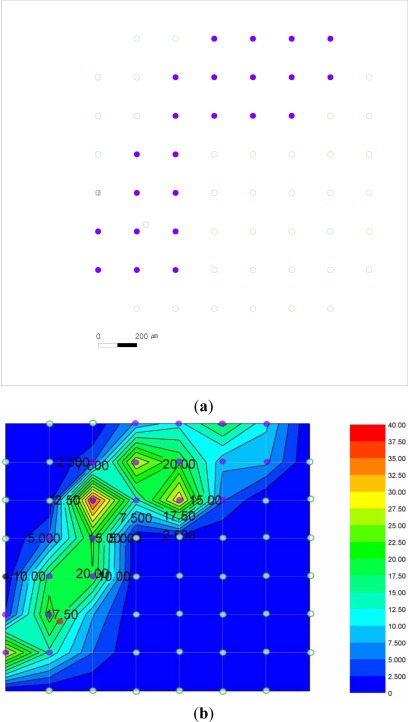

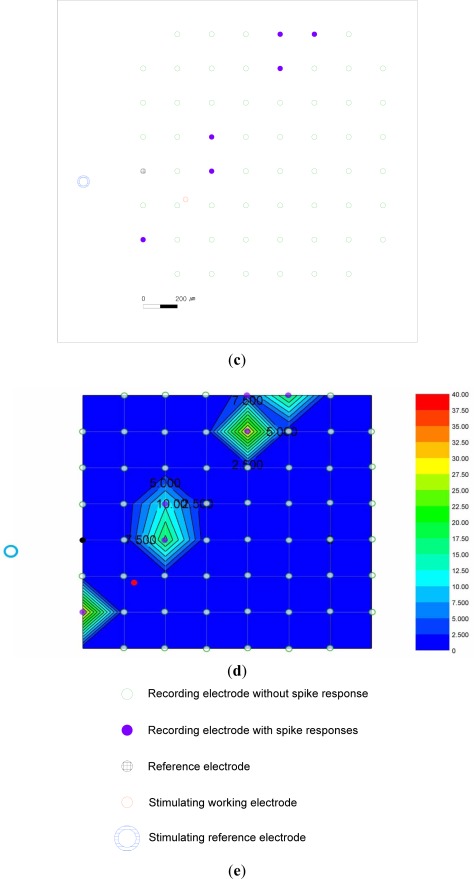
The monopolar stimulation results. (**a**) The spatial response map of the monopolar stimulation. (**b**) The contour diagram of the monopolar stimulation. The numbers on the contour diagram are the evoked RGC spike numbers with respect to 50 μA current stimulation. (**c**) The spatial response map of the bipolar stimulation (**d**) The contour diagram of the bipolar stimulation. The numbers on the contour diagram are the evoked RGC spike numbers with respect to 50 μA current stimulation. (**e**) Electrode indices.

Figure 6 shows the spatial response map of monopolar stimulation (Figure 6(a)) and bipolar stimulation (Figure 6(c)). The bipolar stimulation (Figure 6(c)) is performed immediately after the monopolar stimulation (Figure 6(a)). Therefore, the two experiments have the same microelectrodes locations at the same retinal patch.

In Figure 6(a), the stimulation method is monopolar, and the height from the stimulating MEA to the recording MEA is about 150 μm. The stimulating working microelectrode (red circle with dots) is at the upper and right part near the recording microelectrode of the 6th row and 2nd column 6-2.

The microelectrodes which records the evoked RGC spike (violet solid circle) are the 1–4, 1–5, 1–6, 1–7, 2–3, 2–4, 2–5, 2–6, 2–7, 3–3, 3–4, 3–5, 3–6, 4–2, 4–3, 5–2, 5–3, 6–1, 6–2, 6–3, 7–1, 7–2, and 7–3 microelectrodes. Figure 6(b) shows the contour diagram of the evoked RGC spike number with respect to 50 μA current stimulation of the monopolar stimulation method. The strong responses are distributed between the left lower area and the right upper area.

In Figure 6(c), the stimulation method is bipolar, and the height from the stimulating MEA to the recording MEA is about 150 μm. The stimulating working microelectrode is the same location near the 6–2 microelectrode. The stimulating reference microelectrode (blue donut with lines) is outward from the 1–5 and 1–6 microelectrodes. The microelectrodes which record the evoked RGC spike (violet solid circle) are the 1–5, 1–6, 2–5, 4–3, 5–3, and 7–1 microelectrodes. Figure 6(d) shows the contour diagram of the evoked RGC spike number with respect to 50 μA current stimulation of the bipolar stimulation method. The strong responses are distributed between the left lower area and the right upper area.

### 3.2. Dual Stimulation

**Figure 7.**
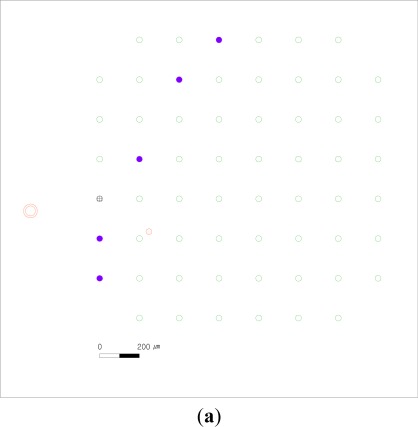

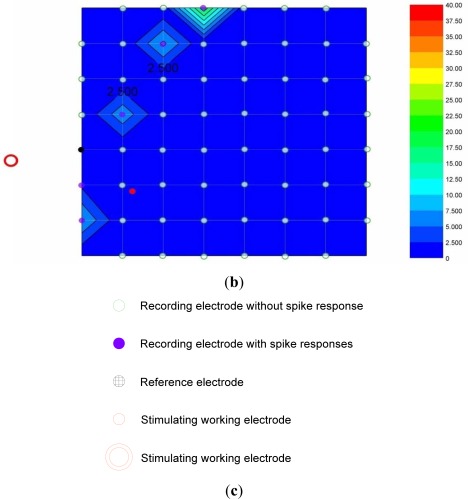
The dual-monopolar stimulation results. (**a**) The spatial response map of the dual-monopolar stimulation. (**b**) The contour diagram of the dual-monopolar stimulation. The numbers on the contour diagram are the evoked RGC spike numbers with respect to 50 μA current stimulation. (**c**) Electrode indices.

Figure 7(a) shows the spatial response map of the dual-monopolar stimulation. The dual-monopolar stimulation (Figure 7(a)) is performed immediately following the bipolar stimulation (Figure 6(c)). Therefore, the three experiments (the monopolar stimulation, the bipolar stimulation and the dual-monopolar stimulation) have the same microelectrodes locations at the same retinal patch. The height from the stimulating MEA to the recording MEA is also about 150 μm. One of the two stimulating working microelectrodes (red circle with dots) is at the upper and right part near the 6–2 microelectrode, and the other of the two stimulating working microelectrodes (red donut with dots) is outward from the 1–5 and 1–6 microelectrodes.

The microelectrodes which record the evoked spikes (violet solid circle) are the 1–4, 2–3, 4–2, 6–1, and 7–1 microelectrodes. This dual-monopolar stimulation shows the smallest number of microelectrodes which record the evoked RGC spikes among all the three stimulation in this research. The strong responses are distributed between the left lower area and the right upper area.

## 4. Discussion

**Figure 8.**
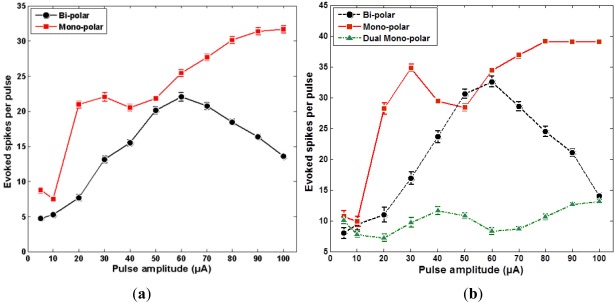
The graph of average number of the evoked RGC spikes. (**a**) Average number of the evoked RGC spikes of the 1–6, 4–3, 5–3, and 7–1 electrodes in both monopolar and bipolar stimulations. (**b**) Average number of the evoked RGC spikes of the 7–1 microelectrodes in all cases.

Results insofar described the data from one patch, however, monopolar and bipolar stimulations were performed for three patches. Ensuing discussions include all 3 set of data. Figure 8(a) show the average number of the evoked RGC spikes of the 1–6, 4–3, 5–3, and 7–1 microelectrodes, which get excited in monopolar and bipolar stimulations. Figure 8(b) show the average number of the evoked RGC spikes of the 7–1 microelectrode, which gets excited in all monopolar, bipolar, and dual-monopolar stimulations. According to Figure 8, the threshold charges of the monopolar and bipolar stimulations are located in the range of 10∼20 nC (which corresponds to 10∼20 μA × 1 ms). However, the threshold charge of the dual-monopolar stimulation method is not clear in the data obtained.

The relation between number of the evoked RGC spikes and electric field intensity is not high. It is considered to be caused by complexity of retinal cell network. This is attributed to the complexity of ganglion cells to bipolar cells network, which is not a simple square grid.

It is remarkable that a similar conclusion was reached in Ahuja, *et al.* which was for *in vitro* epi-retinal configurations [26]. Monopolar, biploar and dual-monopolar stimulations were performed, and the monopolar one showed the most sensitive response with the dual-monoploar showing the least sensitive response. This is a similar conclusion to that obtained from the sub-retinal configuration of this paper. The threshold charge of [26] is 11.6∼36.0 nC, whereas the threshold measured in this paper is 10∼20 nC. These numbers are useful in extracting experimental input ranges, but the numbers cannot be directly compared, since this paper measures ganglion cell response evoked from bipolar cell stimulation in a sub-retinal configuration, and the previous work measures the ganglion cell response from a ganglion cell stimulation in an epi-retinal configuration. Also note that in the direct ganglion cell stimulation to ganglion cell response measurements, the geometric correlation is also stronger.

When the stimulating MEA with the customized jigs dips into the ACSF medium, alignment between the stimulating MEA and the recording MEA is slightly altered. This misalignment is manually corrected by forceps being watched through a microscopy. However, considering recording microelectrode diameter (30 μm), the manual manipulation has its limitation in resolution. Micromanipulators can make more precise alignment. We are adapting micromanipulators for more precise alignment now.

Both the conventional MEA and the developed MEA have two-dimensional microelectrodes. Palanker, *et al.* reported that three-dimensional microelectrodes have advantages of high resolution, low energy consumption, and low tissue damage in a theoretical aspect [31]. Our group also has been developing three-dimensional microelectrodes on polyimide bases for a retinal prosthesis [8]. We are planning to test three-dimensional microelectrode as stimulating microelectrode using the proposed configuration.

## 4. Conclusions

Using two distinct MEA sets, the cell response characteristics in sub-retinal configuration is experimented *in vitro*. An electrically-stimulating MEA is fabricated on a flexible polyimide-substrate using MEMS technology. Retinal patches are placed in between the polyimide-substrate MEA and a commercial glass-substrate MEA. The glass-substrate MEA is used to record retinal ganglion cell responses. The evoked RGC responses of the isolated rabbit retina are recorded for monopolar, bipolar, and dual-monopolar stimulations. Experiments with three retinal patches show that the monopolar excitation showed the most sensitive responses, while the dual-monopolar showed the least sensitive responses. The threshold charges of the monopolar and bipolar stimulation were 10∼20 nC in the sub-retinal *in vitro* experiments.

## Supplementary Materials
