# Supplementary material for: A Novel In Vitro Sensing Configuration for Retinal Physiology Analysis of a Sub-Retinal Prosthesis
Source: Sensors (Basel). 2012 Mar 6;12(3):3131–44. doi: 10.3390/s120303131 (PMC3376591; doi:10.3390/s120303131)
Supplement: Supplementary file 1 [file sensors-12-03131-s001.pdf]

Supplementary Information

A Novel *In Vitro* Sensing Configuration for Retinal Physiology Analysis of a Sub-Retinal Prosthesis

Kyo-in Koo [1], Sangmin Lee [1], Jang Hee Yee [2], Sang Baek Ryu [3], Kyung Hwan Kim [3], Yong Sook Goo [2,*] and Dong-il Dan Cho [1,*]

[1] Inter-university Semiconductor Research Center, Automation System Research Institute, School of Electrical Engineering and Computer Science, Seoul National University, Seoul 151-744, Korea; E-Mails: kkin76@snu.ac.kr (K.K.); sangmlee@snu.ac.kr (S.L.)

[2] Department of Physiology, Chungbuk National University, Cheongju 361-763, Korea; E-Mail: tim1981-yjh@hanmail.net

[3] Department of Biomedical Engineering, Yonsei University, Wonju 220-710, Korea; E-Mails: satisfaction@hanmail.net (S.B.R.); khkim0604@yonsei.ac.kr (K.H.K.)

***** Author to whom correspondence should be addressed; E-Mails: dicho@snu.ac.kr (D.D.C.); ysgoo@chungbuk.ac.kr (Y.S.G.); Tel.: +82-2-880-8371 (D.D.C.); +82-43-261-2870 (Y.S.G.); Fax: +1-805-980-4308 (D.D.C.); +82-43-272-1603 (Y.S.G.).

Received: 15 January 2012; in revised form: 27 February 2012 / Accepted: 27 February 2012 / Published: 6 March 2012

Results of Repeated Experiments

We performed seven experiments (three times of monopolar and bipolar stimulations, and one time of dual-monopolar stimulation) using three retinal patches isolated from one rabbit. In the main paper, one set of representative results is illustrated. In this supplementary file, all results including the representative results (Figure 1) are presented, as shown in Figures 1−4.

Figure 1. The results of the experiment at the retina patch of number 1. (**a**) The spatial response map by the monopolar stimulation at the retina patch of number 1. (**b**) The spatial response map by the bipolar stimulation at the retina patch of number 1. (**c**) The graph of average number of evoked spikes by the monopolar stimulation and bipolar stimulation (the electrodes located in 17, 34, 35, and 61) at the retina patch of number 1. (**d**) The spatial response map by the dual-monopolar stimulation at the retina patch of number 1. (**e**) The graph of comparison among monopolar stimulation, bipolar stimulation, and dual-monopolar stimulation of the electrode located in 17 at the retina patch of number 1.

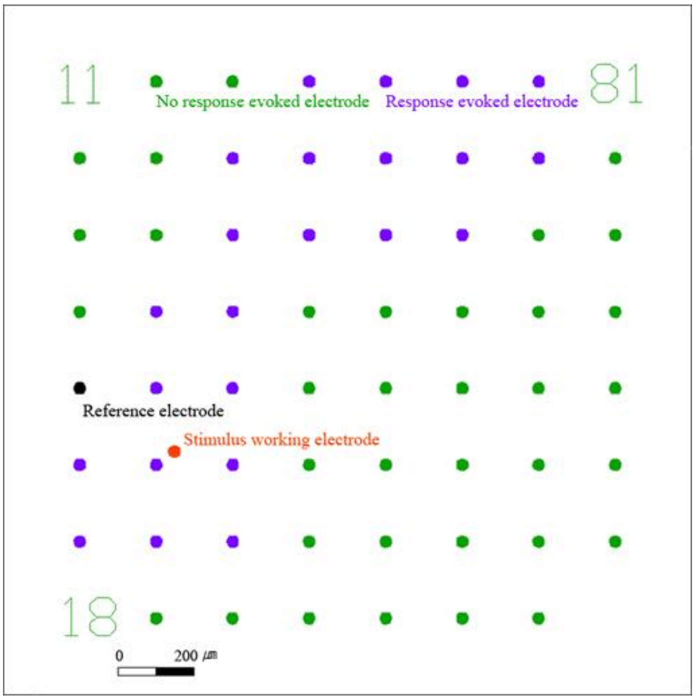

(**a**)

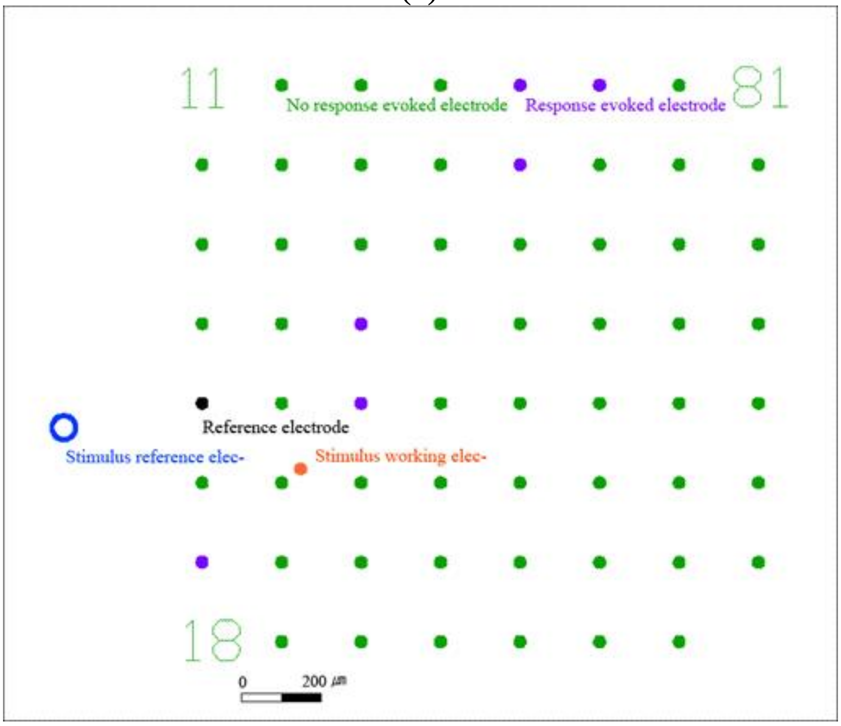

(**b**)

Figure 1. *Cont.*

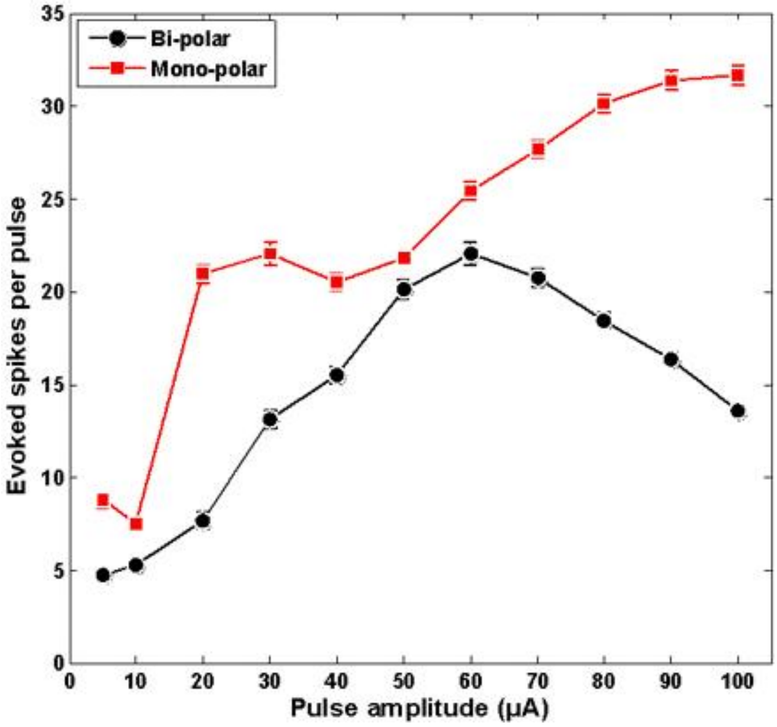

(**c**)

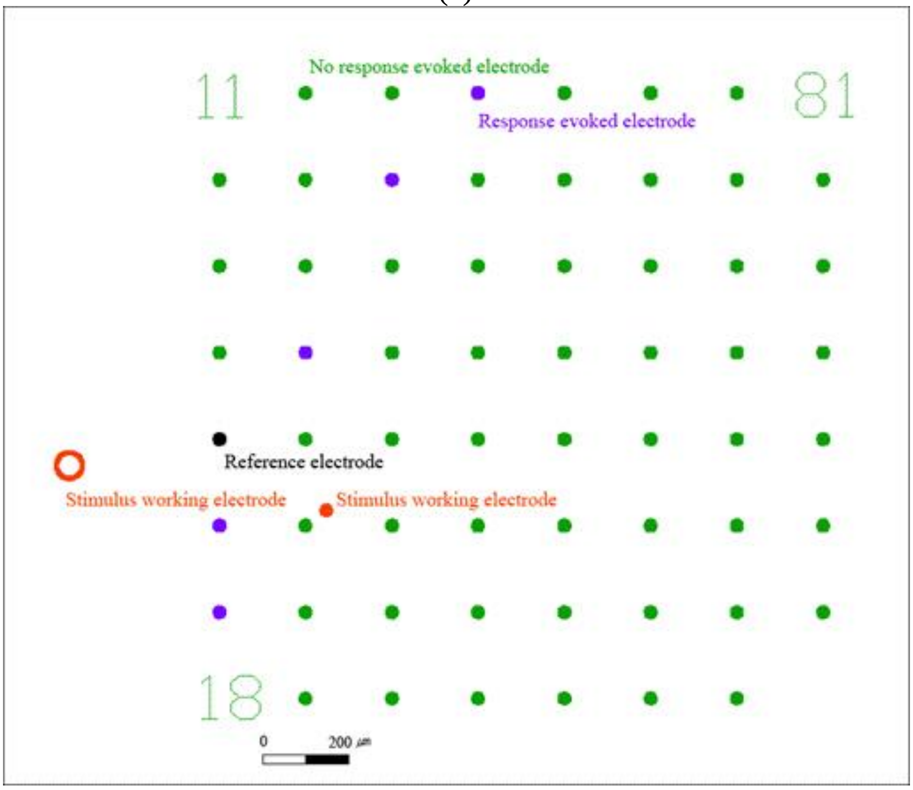

(**d**)

Figure 1. *Cont.*

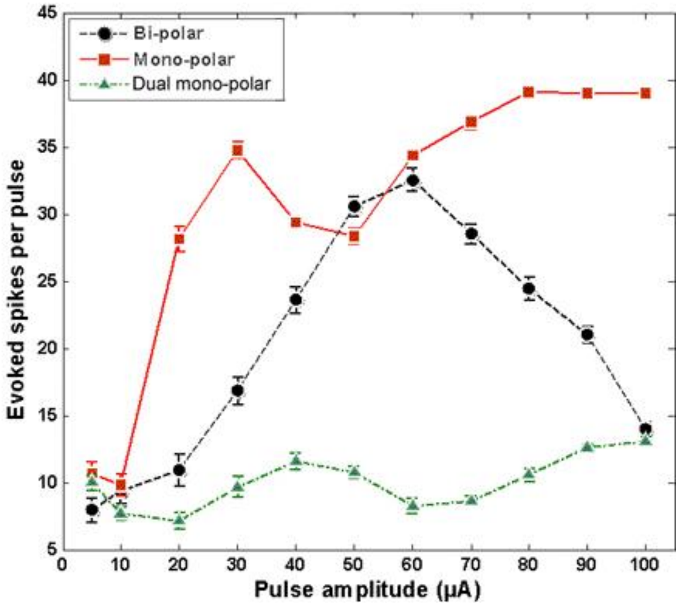

(**e**)

Figure 2. The results of the experiment at the retina patch of number 2. (**a**) The spatial response map by the monopolar stimulation at the retina patch of number 2. (**b**) The spatial response map by the bipolar stimulation at the retina patch of number 2. (**c**) The graph of comparison between monopolar stimulation and bipolar stimulation of the electrode located in 64 at the retina patch of number 2. (**d**) The graph of comparison between monopolar stimulation and bipolar stimulation of the electrode located in 76 at the retina patch of number 2. (**e**) The graph of average number of evoked spikes by the monopolar stimulation and bipolar stimulation (the electrodes located in 65, 66, 67, 68, 76, 77, and 78) at the retina patch of number 2.

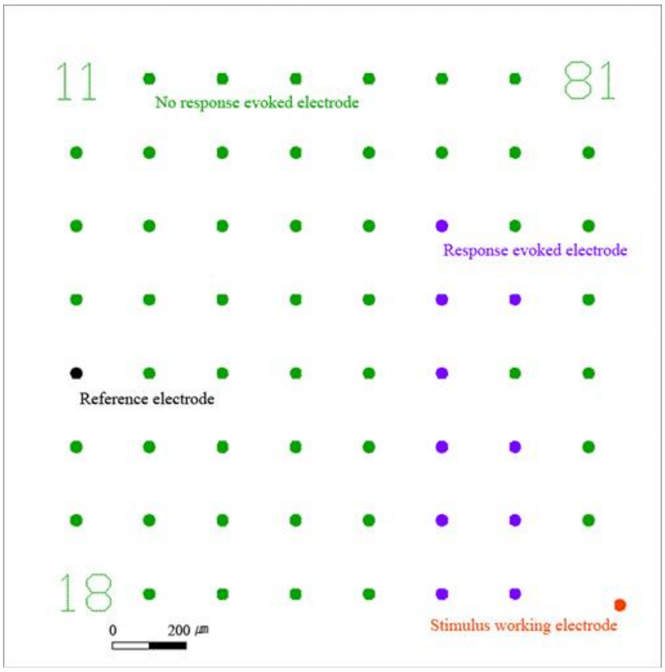

(**a**)

Figure 2. *Cont.*

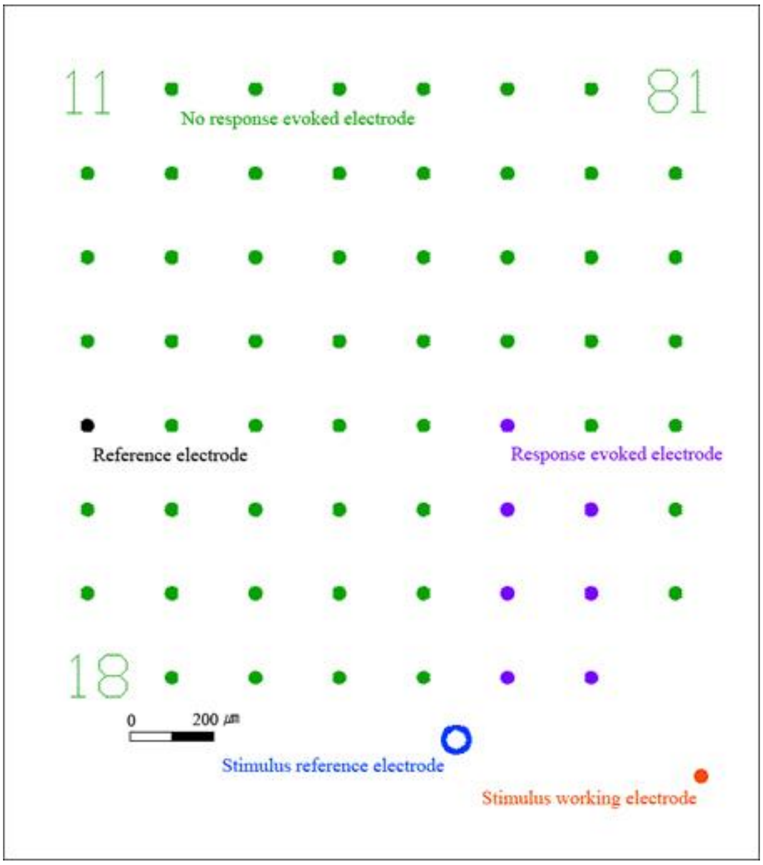

(**b**)

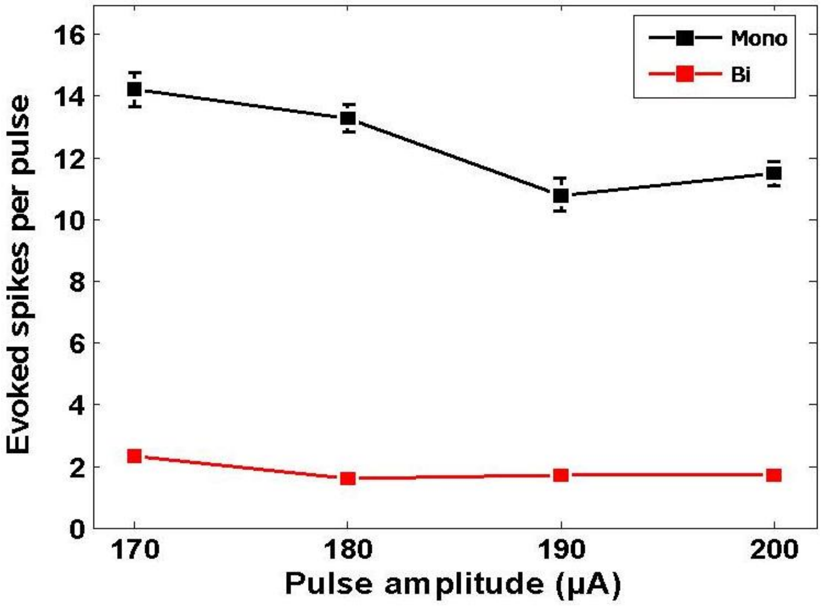

(**c**)

Figure 2. *Cont.*

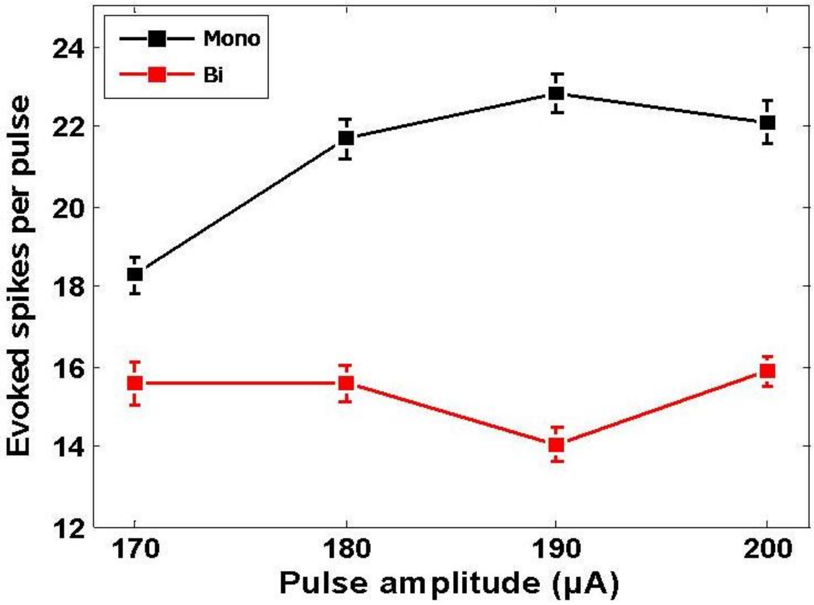

(**d**)

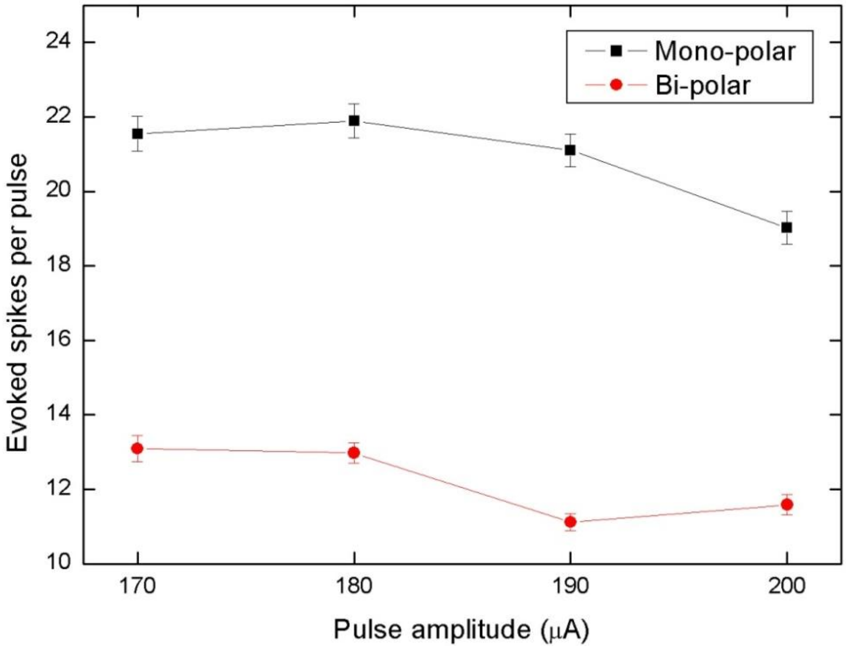

(**e**)

Figure 3. The results of the experiment at the retina patch of number 3. (**a**) The spatial response map by the monopolar stimulation at the retina patch of number 3. (**b**) The spatial response map by the bipolar stimulation at the retina patch of number 3. (**c**) The graph of average number of evoked spikes by the monopolar stimulation and bipolar stimulation (the electrodes located in 25, 35, 36, and 37) at the retina patch of number 3.

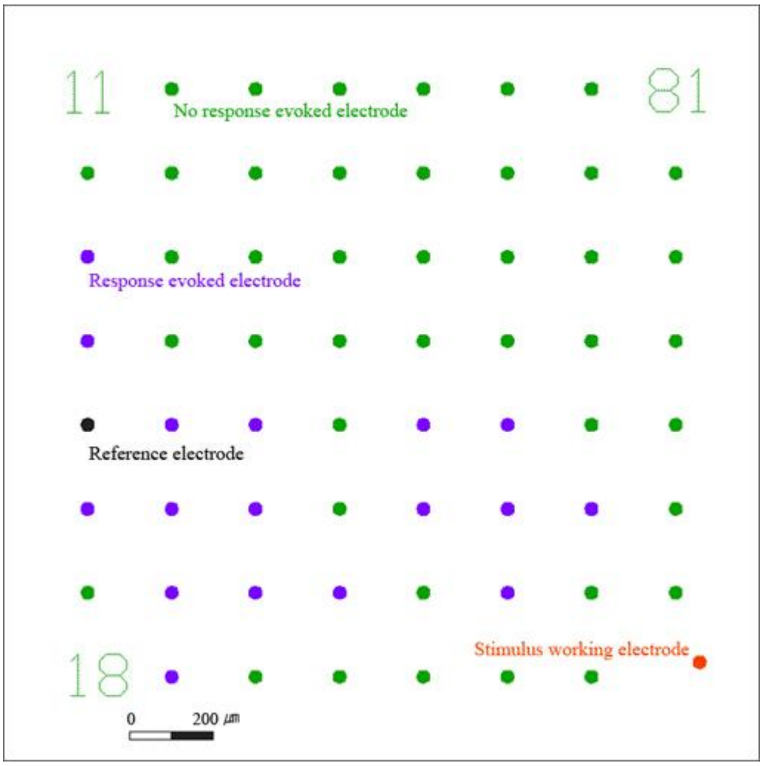

(**a**)

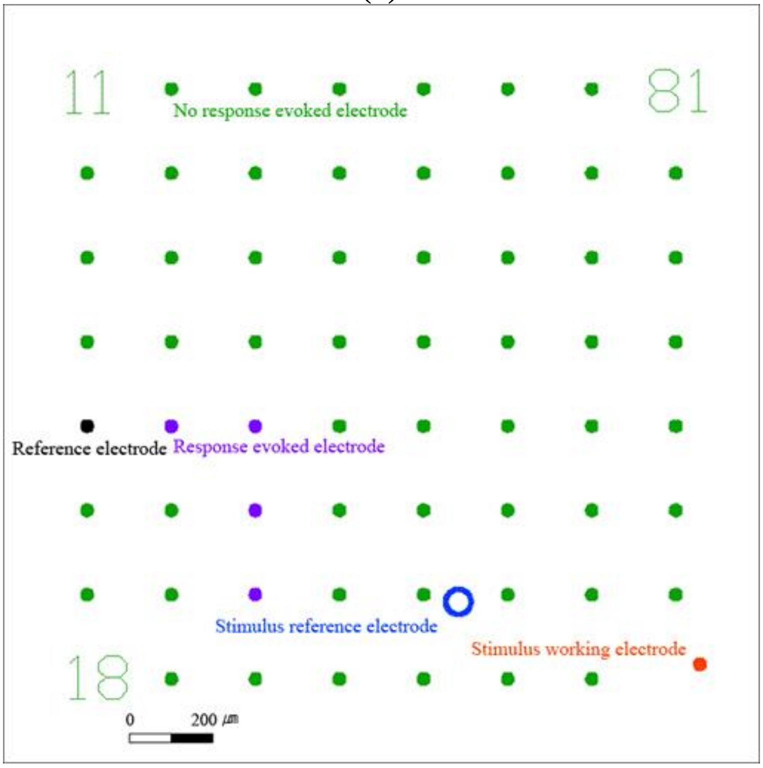

(**b**)

Figure 3. *Cont.*

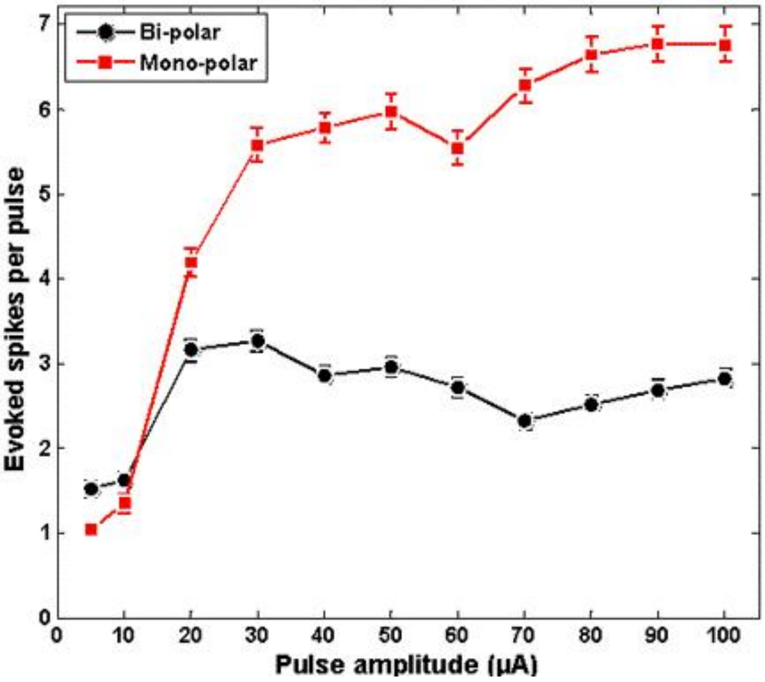

(c)

Figure 4. The graph of average number of evoked spikes by the bipolar stimulation, monopolar, and dual-monopolar stimulation at all the 3 retina patches.

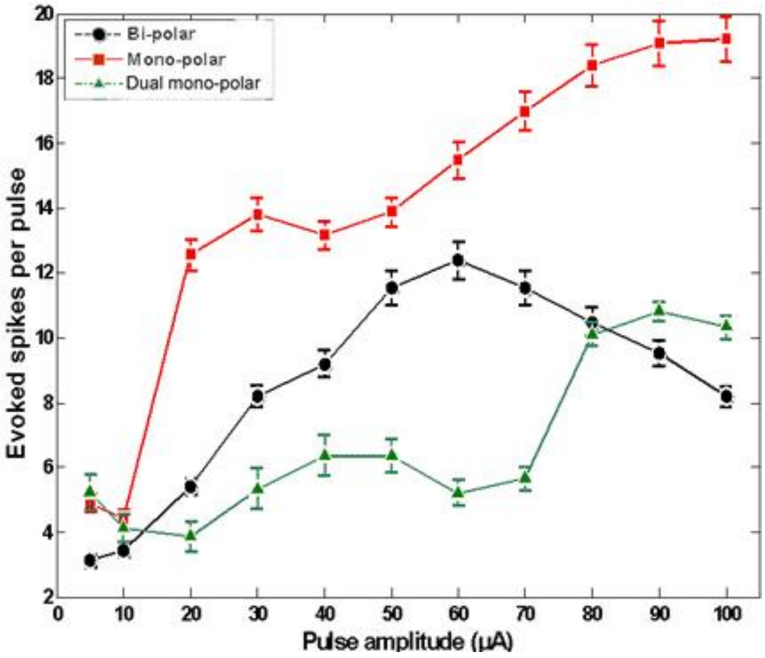

© 2012 by the authors; licensee MDPI, Basel, Switzerland. This article is an open access article distributed under the terms and conditions of the Creative Commons Attribution license (http://creativecommons.org/licenses/by/3.0/).
